# Will there be a critical review on the malignant transformation of oral lichen planus?

**DOI:** 10.1016/j.clinsp.2022.100146

**Published:** 2022-12-05

**Authors:** Dante Migliari

**Affiliations:** Department of Stomatology, Division of Oral Medicine,School of Dentistry, University of São Paulo, São Paulo, Brazil.

Since Oral Lichen Planus (OLP) is a common oral mucosal disease and, possibly, the most common noninfectious oral mucosal disease, its characterization (which includes its variant form ‒ the so-called Oral Lichenoid Lesion [OLL]) as a disease with an intrinsic potential for malignant transformation merits a critical analysis. Whether this characterization should be based on research-based evidence or on loose descriptions of cases is a point at issue. Most cases (supposedly OLP cases) that have undergone malignant transformation have not been subjected to careful analysis, but rather, they were assumed to be OLP lesions by either the authors or (notably) editors and were eventually published as clear-cut cases of malignant transformation of OLP.

For science, this is bad news on the principle that the pervasion of (even unproved) scientific theory takes effect almost immediately; its rectification takes an unpredictable amount of time if it ever occurs. The race theory of inferiority, a Nazi propaganda of tragic consequences, has not been overcome yet. Another example of a disastrous consequence is related to a series of only 12 cases published by the Lancet (1998), which suggested that the Measles, Mumps, and Rubella (MMR) vaccine may predispose recipients to autism. Although the study only included 12 cases, this caused a substantial drop in MMR vaccination rates because parents were concerned about the risk of autism after vaccination. The Lancet later retracted the paper, but by then it was too late.^1^^,^^2^ Several other examples exist, including the opioid epidemic, which stemmed from the false assertion that modern opioid medications were not addictive.

Most cases of malignant transformation of OLP lesions reported in the literature are based on loose evidence, and thus, clarity on the diagnostic process of OLP lesions is lacking. The results of these publications have only generated fear in the population of a clinical outcome of which authors (and editors) are not certain that was fully based on science, that is, whether cases with malignant transformation unequivocally met the criteria for an OLP diagnosis. In one study, a systematic review,^3^ the authors stated that only scattered clinical information could be gathered from articles regarding the OLP patients who developed oral squamous cell carcinoma. Some authors are even more irrational and state that an Oral Lichenoid Contact Reaction (OLCR) to amalgam fillings may result in malignant transformation on the grounds that “unlucky” patients may not have the chance to have their amalgam fillings replaced by a nonmetallic material, since the OLCR lesion resolves after amalgam replacement.

Very few studies have reported well-documented cases of malignant transformation of OLP,^4^^,^^5^ and systematic reviews and meta-analyses have yielded no advances on this issue. On the contrary, they reported (and reproduced) the inconsistency of clinical studies without including any criticism. Additionally, these reviews added some correlations that have no consensus, e.g., stating that hepatitis C virus infection can increase the likelihood of OLP malignancy. However, there is no scientific basis for this assertion. Furthermore, these reviews also make claims based on some loose reports of a new entity termed OLP with dysplasia.^6^^,^^7^ This concept has emerged from a group of authors; unfortunately, once their data were published, the issue of OLP malignancy worsened. Other negative contributions have also been made, including a study on OLP that claimed a lesion may change into carcinoma *in situ*, a “finding” that was also published.^8^

The 1978 WHO report on the diagnostic criteria of OLP^9^ only vaguely mentions the malignant potential of OLP stating “while a number of reports have referred to cancer arising in the erosive or atrophic types of lichen planus, there remains considerable uncertainty about the frequency of this occurrence”. No critical analysis from this WHO report has been performed to determine whether these cases of OLP with malignant transformation were, actually OLP lesions. Although these cases quoted by the WHO were reported by experienced researchers in oral medicine, the authors cannot assume that these researchers were sure, beyond a doubt, that their cases were OLP lesions. Without verifying the accuracy of the data, we enter a world where everyone has his or her own truth.

The real matter of the potential for the malignant transformation of OLP (which has been highlighted by almost all authors) lies in the lack of universally accepted criteria for the diagnosis of OLP. In this scenario, authors feel entitled to establish their own set of criteria for diagnosing OLP, which contributes to a “no man's land” “type of scientific environment”. The editors, for their part, have only enhanced the confusion and uncertainty by their lack of rigor in scrutinizing OLP or OLL cases that underwent a malignant transformation, that is, whether or not the available criteria for OLP diagnosis have been explicitly established and used.

Finally, in the short term, it will not be a feasible endeavor to make advancements in this issue because most editorial boards associated with renowned scientific journals in oral medicine will not change their minds or will not consider that anything has been erroneous with previously published cases asserting that OLP is a true potential malignant oral mucosal disease. This assertion about OLP is not (absolutely) true and requires, fundamentally, additional critical research.

## Conflicts of interest

The author declare no conflicts of interest.
